# Supplementary material for: Preattentive facilitation of target trajectories in a dragonfly visual neuron
Source: Commun Biol. 2022 Aug 18;5:829. doi: 10.1038/s42003-022-03798-8 (PMC9388622; doi:10.1038/s42003-022-03798-8)
Supplement: Supplementary file 2 — Reporting Summary [file 42003_2022_3798_MOESM2_ESM.pdf]

nature portfolio

Corresponding author(s): Benjamin Lancer

Last updated by author(s): 2022/06/29

Reporting Summary

Nature Portfolio wishes to improve the reproducibility of the work that we publish. This form provides structure for consistency and transparency in reporting. For further information on Nature Portfolio policies, see our Editorial Policies and the Editorial Policy Checklist.

Statistics

For all statistical analyses, confirm that the following items are present in the figure legend, table legend, main text, or Methods section.

n/a	Confirmed	
☐	☒	The exact sample size (n) for each experimental group/condition, given as a discrete number and unit of measurement
☐	☒	A statement on whether measurements were taken from distinct samples or whether the same sample was measured repeatedly
☐	☒	The statistical test(s) used AND whether they are one- or two-sided<br>*Only common tests should be described solely by name; describe more complex techniques in the Methods section.*
☒	☐	A description of all covariates tested
☐	☒	A description of any assumptions or corrections, such as tests of normality and adjustment for multiple comparisons
☐	☒	A full description of the statistical parameters including central tendency (e.g. means) or other basic estimates (e.g. regression coefficient) AND variation (e.g. standard deviation) or associated estimates of uncertainty (e.g. confidence intervals)
☐	☒	For null hypothesis testing, the test statistic (e.g. F, t, r) with confidence intervals, effect sizes, degrees of freedom and P value noted<br>*Give P values as exact values whenever suitable.*
☒	☐	For Bayesian analysis, information on the choice of priors and Markov chain Monte Carlo settings
☒	☐	For hierarchical and complex designs, identification of the appropriate level for tests and full reporting of outcomes
☐	☒	Estimates of effect sizes (e.g. Cohen's d, Pearson's r), indicating how they were calculated

Our web collection on statistics for biologists contains articles on many of the points above.

Software and code

Policy information about availability of computer code

Data collection	Data was collected using a custom built software suite based on MATLAB
Data analysis	Data was analyzed using MATLAB including the Wavelet Toolbox

For manuscripts utilizing custom algorithms or software that are central to the research but not yet described in published literature, software must be made available to editors and reviewers. We strongly encourage code deposition in a community repository (e.g. GitHub). See the Nature Portfolio guidelines for submitting code & software for further information.

Data

Policy information about availability of data

All manuscripts must include a data availability statement. This statement should provide the following information, where applicable:

- Accession codes, unique identifiers, or web links for publicly available datasets
- A description of any restrictions on data availability
- For clinical datasets or third party data, please ensure that the statement adheres to our policy

Data/code is available via figshare DOI: 10.25909/19407572

Field-specific reporting

Please select the one below that is the best fit for your research. If you are not sure, read the appropriate sections before making your selection.

☒ Life sciences ☐ Behavioural & social sciences ☐ Ecological, evolutionary & environmental sciences

For a reference copy of the document with all sections, see nature.com/documents/nr-reporting-summary-flat.pdf

Life sciences study design

All studies must disclose on these points even when the disclosure is negative.

Sample size	As attention is a trial-by-trial phenomenon and averaging across trials could mask the effects, we report sample size (n) calculated on the basis of the amount of individual trials. We additionally report the number of dragonflies (one neuron per dragonfly) that were recorded from.
Data exclusions	Although we took measures to avoid neuronal habituation, we also excluded individual trials that exhibited a low spiking response to targets within the excitatory receptive field (Ie, below spontaneous). This measure was not taken for trials involving inhibitory stimuli.
Replication	In order to ensure replication, we repeated both individual trials within an experiment within a single recording multiple times, and repeated entire experiments across multiple dragonflies.
Randomization	The order of presentation of individual trial conditions was randomized for each presentation of the experiment.
Blinding	Individual dragonflies were not assigned to groups, but trial order was blindly randomized using a computer.

Reporting for specific materials, systems and methods

We require information from authors about some types of materials, experimental systems and methods used in many studies. Here, indicate whether each material, system or method listed is relevant to your study. If you are not sure if a list item applies to your research, read the appropriate section before selecting a response.

Materials & experimental systems

n/a	Involved in the study	
☒	☐	Antibodies
☒	☐	Eukaryotic cell lines
☒	☐	Palaeontology and archaeology
☐	☒	Animals and other organisms
☒	☐	Human research participants
☒	☐	Clinical data
☒	☐	Dual use research of concern

Methods

n/a	Involved in the study	
☒	☐	ChIP-seq
☒	☐	Flow cytometry
☒	☐	MRI-based neuroimaging

Animals and other organisms

Policy information about studies involving animals; ARRIVE guidelines recommended for reporting animal research

Laboratory animals	Male Hemicordulia tau (Insecta: Anisoptera)
Wild animals	Wild dragonflies were manually caught with insect nets at the Adelaide Botanic Park and Adelaide Botanic Gardens. Dragonflies were identified (species) and sexed at the field site. Females and teneral males were re-released immediately. After an experiment, all dragonflies were decapitated using scissors and disposed of in a laboratory biological waste bin.
Field-collected samples	Collected dragonflies were stored in a plastic bag with a moist paper towel (to prevent drying) in a dark fridge kept at 7°c for up to 7 days before experimentation.
Ethics oversight	No ethical approval is required for use of dragonflies under the Australian code for the care and use of animals for scientific purposes

Note that full information on the approval of the study protocol must also be provided in the manuscript.
